# The impact of obesity on thyroidectomy outcomes: a case-matched study

**DOI:** 10.1007/s13304-023-01687-1

**Published:** 2023-11-21

**Authors:** Leonardo Rossi, Chiara Becucci, Mattia Iachini, Carlo Enrico Ambrosini, Federica Renieri, Riccardo Morganti, Francesco Pignatelli, Gabriele Materazzi

**Affiliations:** 1 Department of Surgical, Medical and Molecular Pathology and Critical Area, University of Pisa, Pisa, Italy; 2 Section of Statistics, University of Pisa, Pisa, Italy

**Keywords:** Thyroidectomy, Obesity, Complications, Thyroid, Outcomes

## Abstract

Obesity is a well-known public health concern in Western World. Accordingly, an elevated number of obese patients undergo thyroidectomy every year. We aim to assess the impact of obesity on intraoperative and postoperative outcomes of patients who undergo thyroidectomy. 1228 patients underwent thyroidectomy at our department between January 2021 and September 2021. We divided patients into two groups according to body mass index (BMI): non-obese (BMI < 30 kg/m^2^) and obese (BMI ≥ 30 kg/m^2^). A propensity score approach was performed to create 1:1 matched pairs (matching according to age, gender, diagnosis, nodule size and type of operation). After matching, the final population included 522 patients, equally divided between each group: non-obese group (Group A; n = 261) and obese group (Group B; n = 261). The primary endpoint of the study was the overall rate of postoperative complications; secondary endpoints of the study were operative time, use of energy device and length of hospital stay. The duration of hospital stay resulted longer in Group B (p = 0.002). No statistically significant differences were documented in terms of operative time (p = 0.206), use of energy devices (p = 0.855) and surgical complications (p = 0.429). Moreover, no statistically significant differences were documented considering each specific complication: transient and permanent hypocalcemia (p = 0.336; p = 0.813, respectively), transient and permanent recurrent laryngeal nerve palsy (p = 0.483; p = 0.523, respectively), hematoma (p = 0.779), bleeding (p = 0.178), wound infection (p = 0.313) and cheloid formation (p = 0.412). Thyroidectomy can safely be performed in obese patients. Outcomes resulted comparable; nonetheless, obesity correlates to longer hospital stay.

## Introduction

Obesity is a widespread health concern as it is associated with several medical conditions, such as diabetes, hypertension, coronary artery disease, hyperlipidemia and some types of carcinoma [1]. Moreover, obesity leads to a worse quality of life and represents a burdensome factor for health care costs.

Overall, the prevalence of obesity has tripled in the general population between 1975 and 2016 [2]: consequently, more and more obese patients undergo surgical procedures, apart from bariatric surgery itself, including thyroidectomy. Despite considerable investigation, the impact of obesity on thyroidectomy outcomes remains controversial.

We aim to assess the safety of thyroid surgery in obese patients by evaluating the surgical outcomes focusing on postoperative complications.

## Materials and methods

This is a retrospective study performed using a prospectively maintained database. We included all patients aged ≥ 18 years who underwent open thyroid surgery between January 2021 and September 2021. This study was approved by the Internal Research Board.

We divided patients into two groups according to the standardized categories proposed by the World Health Organization: obese (BMI ≥ 30 kg/m^2^) and non-obese (BMI < 30 kg/m^2^) [3].

Demographic, diagnosis, thyroid nodule size, type of surgery, operative time, use of energy device, postoperative hospital stay and postoperative complications (transient and permanent hypocalcemia, transient and permanent recurrent laryngeal nerve (RLN) palsy, cervical hematoma, bleeding which require reoperation, wound infection and cheloid formation) were collected and scrutinized. Operative time was defined from the skin incision to the skin closure. The use of intra-operative nerve-monitoring (IONM) depends on the individual clinical case and the surgeon's preferences.

We defined postoperative hypocalcemia as an albumin-corrected calcium level of < 8.0 mg/dL or inability to interrupt calcium therapy [4]. RLN palsy was diagnosed in case of documented vocal cord mobility alteration at fiberoptic laryngoscopy. We used the cutoff of 6 months to discriminate between transient and permanent postoperative hypocalcemia and RLN palsy.

In order to make groups homogeneous and compare surgical outcomes, minimizing eventual selection biases, two comparable groups were obtained matching 1:1 obese patients with non-obese patients. The following parameter were considered for the matching: age, gender, diagnosis, nodule size, type of surgery. We included non-obese patients in Group A and obese patients in Group B.

The primary endpoint of the study was the overall rate of postoperative complications; secondary endpoints of the study were operative time, use of energy device and length of hospital stay.

### Statistical analysis

Categorical data were described by absolute and relative (%) frequency, continuous data by mean (sd) and median (IQR). Statistical analysis was performed using the Chi-square test for categorical variables and Mann–Whitney test and Student’s *t* test for quantitative variables. To compare more than two groups, Kruskal–Wallis followed by multiple comparisons with Bonferroni method (or one-way ANOVA when appropriate) were used, respectively. Significance was fixed at 0.05. All analyzes were carried out with SPSS v.28 technology (IBM Corp., Armonk, NY, USA).

## Results

Overall, 1228 consecutive patients were submitted to open thyroidectomy within the period analyzed, 261 of whom were obese (21.3%).

After matching, the final population included 522 patients, equally divided between each group: non-obese group (Group A; n = 261) and obese group (Group B; n = 261). The obese and non-obese groups resulted well-matched with respect to age, sex, diagnosis, nodule size and type of surgery (Table 1) as no differences were documented with respect to those variables.

**Table 1:** Propensity score matching; demographic, clinical and surgical variables

	Group A (n = 261)	Group B (n = 261)	P-value
Sex n; (%)			
Male	74 (28.4%)	76 (29.1%)	0.847
Female	187 (71.6%)	185 (70.9%)	
Age (years; mean ± SD)	49.6 ± 15.6	50.2 ± 13.1	0.634
Diagnosis n; (%)			
Benign disease	99 (37.9%)	102 (39.1%)	0.787
Indeterminate	77 (29.5%)	86 (33.0%)	0.395
Malignancy	85 (32.6%)	73 (28.0%)	0.253
Nodule size (mm; mean ± SD)	27.4 ± 16.7	28 ± 16.7	0.661
Surgery n; (%)			
Hemithyroidectomy	49 (18.8%)	46 (17.6%)	0.731
Thyroidectomy	179 (68.6%)	186 (71.3%)	0.446
Thyroidectomy + central neck dissection	13 (5.0%)	11 (4.2%)	0.676
Thyroidectomy + central and lateral neck dissection	20 (7.7%)	18 (6.9%)	0.736

Group A: BMI ≤30 kg/m^2^; Group B: BMI>30 kg/m^2^; SD: standard deviation

Operative outcomes are summarized in Table 2. The median operative time and the rate of use of energy device did not differ between groups (p = 0.206 and 0.855, respectively). No differences were documented regarding the overall postoperative complications rate between groups: 25.3% in the obese group and 28.4% in the non-obese group (p = 0.429). In particular, no statistically significant differences were documented between obese and non-obese groups in terms of postoperative transient (19.5% and 23%, respectively; p = 0.336) and permanent hypocalcemia (4.2% and 4.6%, respectively; p = 0.813) and transient (3.1% and 4.2%, respectively; p = 0.483) and permanent RLN palsy (1.5% and 2.3%, respectively; p = 0.523). Moreover, the rate of cervical hematoma (2.3% in the obese group and 2.7% in the non-obese group; p = 0.779) and bleeding which require reintervention (0.4% in the obese group and 1.5% in the non-obese group; p = 0.178) did not differ significantly. Regarding wound complications, no statistically significant differences were documented in terms of infection (p = 0.313) and cheloid formation (p = 0.412). Lastly, a statistically significant longer hospital stay was documented in the obese group (p = 0.002).

**Table 2:** Operative outcomes

	Group A (n = 261)	Group B (n = 261)	P-value
Operative time minutes; (median range)	50 (40–60)	50 (40–60)	0.206
Energy device n; (%)	170 (65.1)	168 (64.4)	0.855
Hospital stay days; (median range)	1 (1–2)	2 (1–2)	***0.002***
Overall postsurgical complications n; (%)	74 (28.4)	66 (25.3)	0.429
Bleeding n; (%)			
Cervical hematoma	7 (2.7)	6 (2.3)	0.779
Reintervention	4 (1.5)	1 (0.4)	0.178
Hypocalcaemia n; (%)			
Transient	60 (23.0)	51 (19.5)	0.336
Permanent	12 (4.6)	11 (4.2)	0.813
RLN palsy n; (%)			
Transient	11 (4.2)	8 (3.1)	0.483
Permanent	6 (2.3)	4 (1.5)	0.523
Wound complications n; (%)			
Infection	3 (1.1)	6 (2.3)	0.313
Cheloid	4 (1.5)	2 (0.8)	0.412

Group A: BMI ≤30 kg/m^2^; Group B: BMI>30 kg/m^2^; RLN: recurrent laryngeal nerve

71 patients included in Group B were affected by obesity grade II or III (BMI ≥ 35 kg/m^2^): no statistically significant differences in terms of age, sex, diagnosis, nodule size and type of surgery was documented between these patients and those with BMI < 35 kg/m^2^ (obesity grade I) or without obesity (Table 3). Furthermore, no statistically significant difference was documented between Group A and either patients with BMI < or ≥ 35 kg/m^2^ of Group B in terms of overall postsurgical complications (p = 0.595), use of energy device (p = 0.963), operative time (p = 0.219), whereas a statistically significant difference was found in terms of length of hospital stay (p < 0.001). After multiple comparison with Bonferroni method, the difference remains significant either between Group A and obesity grade I (p < 0.001) and Group A and obesity grade II and III (p = 0.018) (Table 4).

**Table 3:** Demographic, clinical and surgical variables

	Group A (n = 261)	Group B (n = 261)		p-value
	No Obesity Group (n = 261)	Obesity grade I (n = 190)	Obesity grade II and III (n = 71)	
Sex				
Male n; (%)	74 (28.4)	61 (32.1)	15 (21.1)	0.214
Female n; (%)	187 (71.6)	129 (67.9)	56 (78.9)	
Age (years; mean ± SD)	46.8 ± 15.6	51.5 ± 12.7	46.8 ± 13.6	0.055
Diagnosis				
Benign disease n; (%)	99 (37.9)	78 (41.1)	24 (33.8)	0.590
Indeterminate n; (%)	77 (29.5)	62 (32.6)	24 (33.8)	
Malignancy n; (%)	85 (32.6)	50 (26.3)	23 (32.4)	
Nodule size (mm; mean ± SD)	27.4 ± 16.7	28.9 ± 17.5	25.7 ± 14.4	0.353
Surgery				
Hemithyroidectomy n; (%)	49 (18.8)	32 (16.8)	14 (19.7)	0.852
Thyroidectomy n; (%)	179 (68.6)	138 (72.7)	48 (67.6)	
Thyroidectomy + central neck dissection n; (%)	13 (4.9)	9 (4.7)	2 (2.8)	
Thyroidectomy + central and lateral neck dissection n; (%)	20 (7.7)	11 (5.8)	7 (9.9)	

Group B patients were divided on the basis of the obesity grade

Group A: BMI ≤ 30 kg/m^2^; Group B: BMI > 30 kg/m^2^; Obesity grade I: BMI < 35 kg/m^2^; Obesity grade II and III: BMI ≥ 35 kg/m^2^; SD: standard deviation

**Table 4:** Operative outcomes

Outcomes	Group A (n = 261)	Group B (n = 261)		p. value
	No Obesity Group (n = 261)	Obesity grade I (n = 190)	Obesity grade II and III (n = 71)	
Operative Time minutes; (median range)	50 (40–60)	55 (45–65)	50 (40–60)	0.219
Hospital stay Days; (median range)	1 (1–2)	2 (1–2)	2 (1–2)	< 0.001*
Energy devices n; (%)	170 (65.1)	123 (64.7)	45 (63.4)	0.963
Overall postsurgical complications n; (%)	74 (28.4)	46 (24.2)	20 (28.2)	0.595
Hypocalcemia n; (%)				
Transient n; (%)	60 (22.9)	35 (18.4)	16 (22.5)	0.484
Permanent n; (%)	12 (4.6)	8 (4.2)	3 (4.2)	0.983
RLN palsy n; (%)				
Transient	11 (4.2)	7 (3.6)	1 (1.4)	0.534
Permanent	6 (2.3)	4 (2.1)	0 (0)	0.443
Bleeding n; (%)				
Cervical hematoma	7 (2.7)	4 (2.1)	2 (2.8)	0.911
Reintervention	4 (1.5)	1 (0.5)	0 (0)	0.374
Wound complications n; (%)				
Infection	3 (1.2)	4 (2.1)	2 (2.8)	0.557
Cheloid	4 (1.5)	2 (1.1)	0 (0)	0.555

Group B patients were divided on the basis of the obesity grade

^*^Significant multiple comparisons with Bonferroni method: No Obesity vs Grade I Obesity: p < 0.001—No Obesity vs Grade II + III Obesity: p = 0.018

Group A: BMI ≤ 30 kg/m^2^; Group B: BMI > 30 kg/m^2^; Obesity grade I: BMI < 35 kg/m^2^; Obesity grade II and III: BMI ≥ 35 kg/m2; RLN: recurrent laryngeal nerve

## Discussion

The prevalence of obesity and the number of obese patients scheduled for surgical operation is alarming increasing. It has been previously reported that obesity may negatively influence the rate of surgical complications [5]. Several studies documented a higher complications rate in obese patients compared to non-obese [6–8]; nonetheless, an unanimous opinion is still lacking [9, 10].

Endocrine surgeons are increasingly faced with patients with elevated BMI. Obese patients usually presented a short and large neck with limited possibility of hyperextension, leading to reduced operatory space and consequently hard surgery [11, 12]. Some surgeons considered these features as related to a higher complications rate and longer operative time. We aim to assess the impact of obesity on thyroidectomy outcomes, in particular on postoperative complications.

Our study demonstrated that post-surgical complications of obese patients are comparable to those of non-obese: no statistically significant differences in terms of transient and definitive hypocalcemia, transient and definitive RLN palsy, hematoma, bleeding that requires reoperation and wound complications (infection and cheloid formation) were documented. These findings were confirmed even comparing non-obese patients with those with BMI ≥ 35 kg/m^2^ (obesity grade II and III).

Buerba et al. [13] in 2011 published a multi-institutional study in order to assess the 30-day clinical and economic outcomes of patients scheduled for endocrine neck surgery on the basis of BMI. The authors enrolled 18,825 patients from the American College of Surgeons National Surgery Quality Improvement Program database and documented that obese patients were associated to a higher rate of overall and wound complications. Moreover, obese patients were associated to longer operative time. However, the authors did not assess the rate of endocrine-specific complications, such as RLN palsy and hypocalcemia, limiting the power of the study [13].

Tresallet et al. [14] performed a retrospective study focusing on 1216 papillary thyroid carcinoma patients who underwent thyroid surgery. The authors found an association between recurrent/residual thyroid cancer and BMI, suggesting that obese patients may correlate with more difficult operation. Furthermore, although no significant differences were documented in terms of overall rate of complications, the rate of RLN palsy was higher in the obese group.

Recently, Jin et al. [11] assessed the impact of BMI on complication rate in patients with papillary thyroid cancer and lateral neck metastasis. The authors found that obesity was associated to a higher risk of postoperative bleeding, accessory nerve injury, infection and longer operative time. Nonetheless, the BMI cut-off considered to define obesity was 28.0 kg/m^2^, rather than 30.0 kg/m^2^. Similarly, a previous study on 2678 patients found obesity as a factor which significantly increase the risk of neck hematoma [15].

Notwithstanding, the issue of obesity is debated and several studies reported no significant influence on postoperative outcomes. Farag et al. [16] in a retrospective 3-year study reported no statistically significant differences in terms of postoperative complications, operative time and hospital length of stay between obese and non-obese patients.

Similarly, Canu et al. [17] recently published a large retrospective study on 813 patients (135 of whom were obese) and documented no statistically significant differences regarding postoperative complications and length of hospital stay; nonetheless, the authors reported a significant longer operative time in patients with BMI ≥ 30.0 kg/m^2^. Other studies [12, 18] reported superimposable findings regarding overweight and non-overweight patients (BMI < or ≥ 25.0 kg/m^2^).

Our study mirrors these reports, claiming thyroidectomy in obese patients as a safe and effective procedure regardless the obesity grade. Nonetheless, the lack of statistically significant differences in terms of surgical outcomes (postsurgical complications, operative time, use of energy devices) for obese patients may be due to the large number of patients with high BMI operated every year at our Institution, which made surgeons skilled in this cohort of patients.

On the other hand, as previously reported by Harari et al. [19], we documented a longer length of hospital stay in obese patients. This finding may be attributed to the fact that obese patients are often affected by several comorbidities (such as cardiovascular diseases, respiratory diseases, diabetes mellitus) which require post-operative monitoring in the intensive care unit, prolonging the length of the hospital stay and consequently influencing the hospital resources. Thyroidectomy is an operation which usually requires a short postoperative course which may significantly increase in case of extension of 1 or 2 days.

The obese body habitus was initially seen with great concern when a minimally-invasive or remote-access thyroidectomy was scheduled [20, 21]. Nonetheless, several studies reported no statistically significant differences in terms of surgical complications in case of minimally-invasive video-assisted thyroidectomy (MIVAT), robotic bilateral axillo-breast approach thyroidectomy and robotic transoral thyroidectomy, claiming these approaches as safe and effective in patients with elevated BMI [22–24]. However, it is noteworthy to underline that some case series took into account a limited number of obese patients.

In a recent study, Yap et al. assess the impact of BMI on robotic transaxillary thyroidectomy [25]. Overall, the authors enrolled 3697 patients, 559 of whom were overweight and 76 of whom were obese. After multivariate analysis, the authors found that seroma formation, transient voice hoarseness and operative time were related to increasing BMI [25]. These findings suggest that, although obesity should not be considered a contraindication to robotic transaxillary thyroidectomy, this cohort of patients require an elevated expertise in the field of robotic and endocrine surgery.

Our study harbors several limitations. First of all, the retrospective nature of the paper. Moreover, the enrolled patients underwent different surgical procedures (total thyroidectomy, thyroid lobectomy, total thyroidectomy and central neck dissection, total thyroidectomy and lateral neck dissection) which may have introduced some potential bias. However, in order to attenuate these limitations, we use a case–control matching methods to make the compared groups homogenous in terms of surgical intervention, as well as age, sex, preoperative diagnosis and nodule size. This approach leads to the creation of two comparable groups, minimizing the risk of potential bias. In addition, although the sample size is greater than the major part of previously published papers, because of the low rate of post-operative complications, some outcomes may not have reached the adequate statistical power to highlight significant differences. Moreover, these data come from a high-volume Institutions and may not be reproducible in all centers.

In conclusion, in high-volume Institutions, thyroid surgery in obese patients is safe and not associated to worse postoperative outcomes. It correlates to longer postoperative hospital stay which is mainly related to the immediately postoperative course in intensive care unit due to comorbidities. Future multicentric studies on larger cohorts are required to confirm these findings.

## Data Availability

Data are available upon reasonable request.
